# Agricultural drought monitoring and early warning at the regional scale using a remote sensing-based combined index

**DOI:** 10.1007/s10661-024-13265-y

**Published:** 2024-10-30

**Authors:** Trupti Satapathy, Jörg Dietrich, Meenu Ramadas

**Affiliations:** 1 School of Infrastructure, IIT Bhubaneswar, Odisha, 752050 India; 2 Institute for Hydrology and Water Resources Management, Leibniz Universität Hannover, Hanover, Germany; 3 School of Infrastructure, IIT Bhubaneswar, Odisha, 752050 India

**Keywords:** Agricultural drought, Remote sensing, Shannon’s entropy method, Drought hotspot, Drought early warning

## Abstract

Early detection of agricultural drought can alert farmers and authorities, enhancing the resilience of the food sector. A framework is proposed for developing a novel regional agricultural drought index (RegCDI) by combining remotely sensed vegetation health, soil moisture and crop water stress via a transparent Shannon’s entropy weighting method. The framework consists of the selection of suitable datasets based on their regional performance, the aggregation of selected drought indicators, the validation of the combined index against crop yield, and the testing of predictive capabilities. The creation and performance of RegCDI are demonstrated for the drought prone Indian state of Odisha. MODIS surface reflectance is selected for crop water stress and GLDAS-2 for assessing soil moisture deficits and vegetation health. Three selected indicators (SMCI, TCI, and SIWSI-1) are combined into RegCDI for Odisha. The performance of RegCDI is evaluated (a) against other popular drought indices and (b) by comparing with seasonal crop yields. RegCDI is used to identify drought hotspots based on drought severity, duration, and propensity over the study area. A reforecast evaluation of RegCDI (up to three months ahead) showed that the indicators based on soil moisture deficit and crop water stress could predict drought conditions up to two months ahead with no less than 80% accuracy. This demonstrated the potential of the RegCDI framework and its component indicators for early warning of drought in Odisha.

**Supplementary Information:**

The online version contains supplementary material available at 10.1007/s10661-024-13265-y.

## Introduction

Drought, a natural hazard caused by water deficit, is characterized by a slow onset and gradual spatiotemporal evolution. Monitoring drought characteristics (severity, duration, and return period) is crucial due to its widespread impact and has been a research challenge for decades (Dai et al., 2020). The definition of drought and its different types: meteorological, agricultural, hydrological (surface and/or groundwater), and socio-economic droughts, are well documented (Mishra & Singh, 2010). Numerous studies outline the negative impacts of droughts on crop productivity, socio-economic development, regional and global economies, and ecological health (Henchiri et al., 2020; Wilhite et al., 2007). The primary cause of drought events is precipitation deficits compared to normal patterns (Gimeno-Sotelo et al., 2024). The propagation from meteorological drought to agricultural drought manifests in reduced soil moisture levels when compared to normal values, and further, it also leads to hydrological droughts in the region (Zhang et al., 2021b). In particular, agricultural droughts can significantly weaken the economic development and productivity of countries around the world. India is an agrarian country where the agricultural sector accounted for 17.6 to 20.2% of gross value added (GVA) during 2012–2021 (Economic Survey 2021–2022, Government of India). During the period 1870–2016, India experienced multiple 5 to 17-year droughts, resulting in famine and mass human and livestock losses. The drought of 1918 affected over 65% of the country (Mishra et al., 2019b). There is a clear need for continuous monitoring of agricultural droughts and exploration of different approaches for early drought warning in the region. Few recent studies have combined different drought indicators for monitoring in different parts of India, e.g., Prajapati et al. (2022), Dilip et al. (2023) and Saini & Singh (2024).

Agricultural drought severity quantification varies across studies (Fensholt & Sandholt, 2003; Mishra & Singh, 2010; Samantaray et al., 2019). Popular drought indicators corresponding to vegetation health (green cover, biomass), soil moisture levels (surface and root zone soil moisture), crop stress status (canopy stress, crop water stress), and hydroclimatic variables (land surface temperature, precipitation, evapotranspiration, streamflow, and groundwater level), as well as their combinations, could be identified as useful for effective agricultural drought monitoring (Kogan, 1995; Narasimhan & Srinivasan, 2005; Mishra et al., 2015; Zhang et al., 2021a). Popular indices include the normalized difference vegetation index (NDVI; Townshend et al., 1985; Tucker & Sellers, 1986), the normalized difference water index (NDWI; Gao, 1996), the vegetation condition index (VCI; Liu & Kogan, 1996), the temperature condition index (TCI; Kogan, 1995), the vegetation health index (VHI; Kogan, 1995), the vegetation temperature condition index (VTCI; Wang et al., 2001), and the drought severity index (DSI; Zhang & Yamaguchi, 2014) for vegetation health and the soil water deficit index (SWDI; Martínez-Fernández et al., 2016; Mishra et al., 2017), the soil moisture deficit index (SMDI; Narasimhan & Srinivasan, 2005), and the soil moisture condition index (SMCI; Zhang & Jia, 2013) for soil moisture.

Multivariate drought indices have also been proposed, such as the scaled drought condition index (SDCI; Rhee et al., 2010) and the soil moisture agricultural drought index (SMADI; Sánchez et al., 2016). For the aggregation of multiple variables into a combined or composite index, several techniques have been applied. Dilip et al. (2023) used sets of predefined weights.

Seyedabadi et al. (2020) used copula probability distributions. Among the most used techniques are principal component analysis (PCA) and entropy weighting. PCA reduces the dimensionality of the original data by transforming the original variables into independent principal components. Karimi et al. (2022) showed that the first principal component explained more than 80% of the total variance of the matrix of the investigated indices. Prajapati et al. (2022) used PCA but derived weights for the variables from the eigenvalues of the principal components. The entropy weighting method based on the theory of information entropy by Shannon (1948) directly provides weights of the variables according to their informational content, in particular, according to the contribution of the single variables to the uncertainty of the combined index. It had numerous applications in integrated hydrological-agricultural studies, such as those by Zou et al. (2006), Zhu et al. (2018), and Seyedabadi et al. (2020).

Recent studies have developed drought monitoring frameworks using remote sensing datasets (precipitation, land surface temperature, surface reflectance, and soil moisture) from different satellites and sensors (Son et al., 2012). Drought monitoring using remote sensing datasets has gained popularity in recent decades because: (i) drought variables such as soil moisture, evapotranspiration, temperature, and vegetation health are not always available as measured data for large areas, and (ii) synoptic-scale monitoring is possible over the study area using remote sensing satellites. Continuous high-resolution datasets have become more accessible due to recent advances in remote sensing. Remotely sensed datasets could be used as proxies for vegetation health, representing vegetation growth and productivity, soil moisture deficits, and crop stress, in the absence of continuous ground measurements of these variables (Ghulam et al., 2008; Khanna et al., 2007; Kogan, 1995). Many drought studies used soil moisture and hydrological data from remote sensing (Cui et al., 2021; Mishra et al., 2017; Nicolai-Shaw et al., 2017), like Advanced Microwave Scanning Radiometer-Enhanced (AMSR-E; Kawanishi et al., 2003), the Soil Moisture and Ocean Salinity (SMOS; Kerr et al., 2001) mission, the Advanced Scatterometer (ASCAT; Bartalis et al., 2007), the Soil Moisture Active and Passive (SMAP; Entekhabi et al., 2010) mission, and the Sentinel I (Bauer-Marschallinger et al., 2018; Dilip et al., 2023). As discussed above, vegetation indices derived from satellite observations such as NDVI, VCI, VHI, and NDWI, among others, are useful for drought monitoring at global and regional scales (Sharara et al., 2022; Xie & Fan, 2021). Comprehensive reviews of the characteristics, analysis, and assessment of agricultural droughts using advanced remote sensing tools were provided by Sivakumar et al. (2011) and Mullapudi et al. (2023). Numerous studies have used different remote sensing based data for agricultural drought monitoring in India (Chattopadhyay et al., 2020; Sandeep et al., 2021; Vyas et al., 2015).

Monitoring agricultural drought at a regional scale by integrating the most relevant drought indicators that can reflect vegetation health, crop water stress, and soil moisture levels is advantageous (AghaKouchak et al., 2015; West et al., 2019), but its validation can be challenging for the research community. For the practical use of drought indices in policy making to be successful, it is necessary to validate the drought monitoring framework with parameters such as observed yield. It is imperative that drought occurrence is simultaneously well correlated with decreases in crop production and yield. Site-specific analysis of drought indicators could also better reflect how their relative importance and agricultural drought characteristics vary spatially.

Agricultural drought indices are often used for retrospective assessments of droughts, which helps to improve the knowledge of droughts and their interactions with other factors. However, their potential for practical application for the benefit of society lies in the early detection of droughts and the provision of warnings, ultimately to increase the resilience of communities vulnerable to the predicted droughts. There are limited applications where a drought monitoring framework is being developed with the potential to suggest drought hotspots as well as for early drought warning.

The objective of this study is to develop and validate a framework for drought monitoring and prediction using remote sensing datasets. Existing indices used to detect prevailing drought conditions use information on vegetation conditions, soil moisture status, etc., individually, which may not adequately represent the actual drought phenomenon at the regional scale. Conversely, agricultural drought is the combined effect of poor vegetation conditions, stressed vegetation and low soil moisture conditions. Therefore, to detect prevailing agricultural drought conditions more accurately and precisely, the present study combined these prevailing drought factors from easily accessible sources to perform synoptic-scale drought assessment and monitoring with the utmost accuracy.

The novel agricultural drought monitoring framework proposed for regional agricultural drought monitoring integrates different drought indicators derived from remote sensing satellite datasets and land surface models. The article demonstrates how a combined regional drought index is developed from the raw information available on the potential drought indicator variables, explaining the rationale for selecting only the key predictors and integrating information from different predictors, with scope for validating the performance of the index. The drought variables are selected to represent the effects of vegetation health, soil moisture deficit, and crop water stress based on their associations with parameters such as yield and meteorological drought in the region. The joint index is calculated using weights obtained from Shannon’s entropy method. The proposed index, the regional combined drought index (RegCDI), is then used to monitor agricultural drought and identify drought hotspots in the study area. Furthermore, the drought early warning capabilities of the proposed framework are evaluated at different lead times ranging from 1 to 3 months, which can improve its practical use for the agricultural community.

## Materials and methods

### Study area

**Fig. 1:**
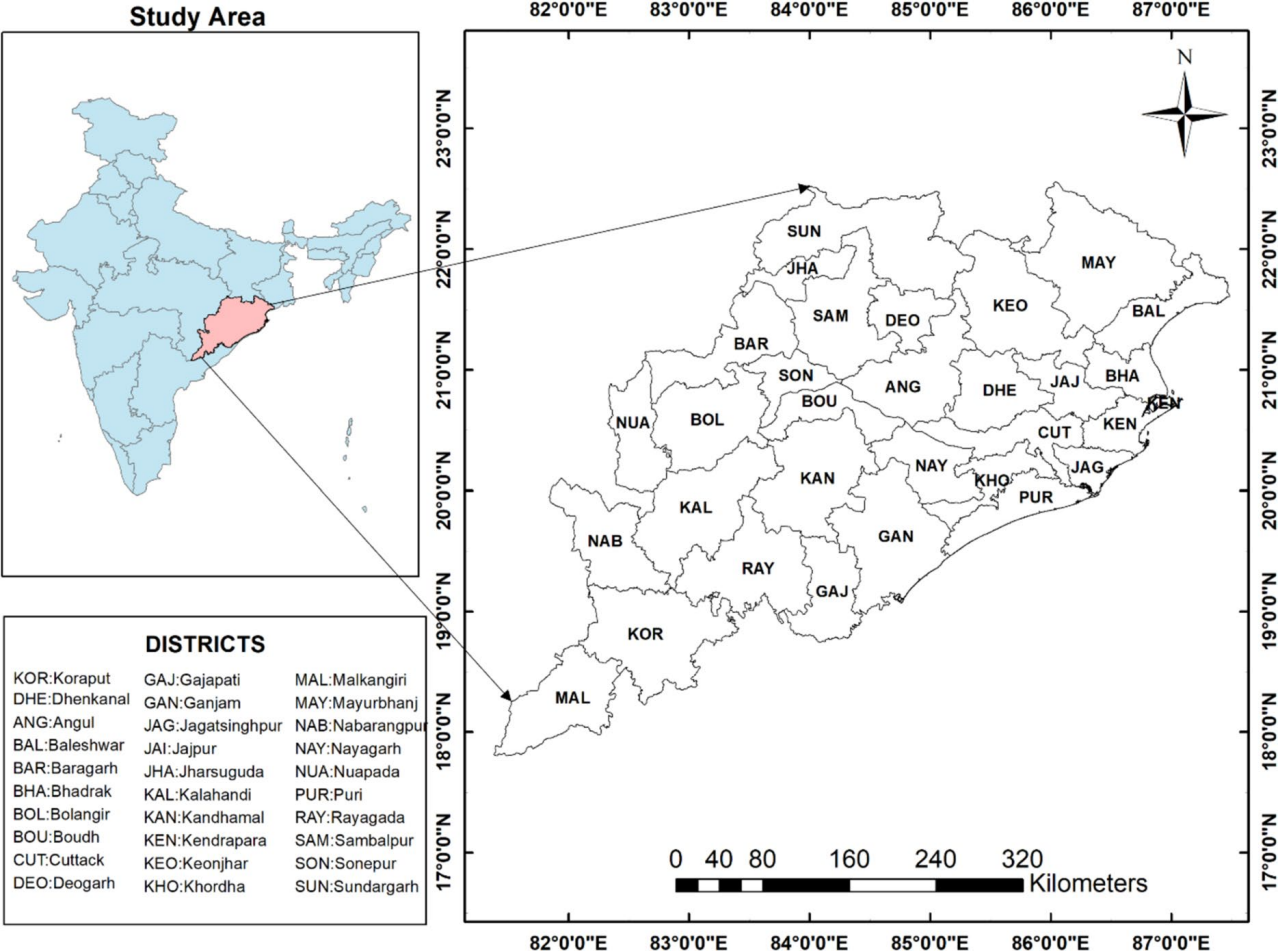
Map of the study area: the State of Odisha in India and its 30 districts

The study area chosen for this study is the state of Odisha in India, which is located between 17.49°N and 22.34°N latitude and between 81.27°E and 87.29°E longitude with a long coastline on the Bay of Bengal as shown in Fig. 1.

Odisha has 30 districts (see map in Supplementary Figure S1) and a total area of 155,707 square kilometers. The climate of the region is characterized by hot summers, high rainfall, and cool winters (tropical monsoon weather). While the average temperature varies between 25 °C and 36 °C, the normal rainfall in Odisha of monsoon is 1150.2 mm (mausam.imd.gov.in). There are three major cropping seasons, namely, summer (March to June), monsoon, popularly known as Kharif (where sowing takes place in June/July and harvesting is done in September/October), and winter or Rabi (where sowing takes place in October/November and harvesting is done in January/February). Approximately 60% of the population of Odisha depends on agriculture as the source of income, and the main crop grown in this region is paddy (rice), specifically Kharif paddy rice (DA&FP, 2020). Rain-fed paddy cultivation in water-stressed pockets of Odisha has faced yield losses in years with below-normal rainfall. Crops grown in this region include cereals (maize, wheat, jowar, and millets) and pulses (peas, lentils) among others (agri.odisha.gov.in, DA&FP, 2020). However, the region is highly prone to natural disasters such as droughts, floods, and cyclones, and numerous instances of drought in recent decades have adversely affected Odisha’s productivity according to DA&FP (2020). The mild to severe category of agricultural droughts in Odisha has been attributed by some studies to low soil moisture (Das et al., 2020). Recently, the frequency of drought has been reported to be once in three years, with cropland accounting for 54.57% of the total area (Mukherjee & Hazra, 2022). Studies by Samantaray et al. (2019) and Mishra et al., (2019a, b) reported a significant decrease in yield and increase in crop loss in the Odisha region, which is likely due to droughts.

### Data

In this study, satellite and sensor-based datasets of land surface temperature, surface reflectance, and soil moisture content from various sources such as the National Aeronautics and Space Administration (NASA) Global Land Data Assimilation System Version 2 (GLDAS-2), the Famine Early Warning Systems Network (FEWS NET) Land Data Assimilation System (FLDAS), the LANDSAT, and the Moderate Resolution Imaging Spectroradiometer (MODIS) are retrieved and analyzed (details are provided in Table 1). The different drought indicators useful for agricultural drought monitoring are listed in Table 2. The most suitable datasets for the calculation of drought indicators are selected using the methodology described in the Appendix. In this study, the NDWI, VCI, and TCI are evaluated for monitoring vegetation conditions. The following soil moisture level indicators are adopted: the SMCI, SMDI, and SWDI. The shortwave infrared water stress indices SIWSI-1 and SIWSI-2 are used in this study to represent crop water stress, and they differ in the choice of bands for measuring surface reflectance (Fensholt & Sandholt, 2003). For example, SIWSI-1 is calculated by using band 2 and band 5 of spectral reflectance data set. According to Fensholt & Sandholt (2003), the SIWSI indices can detect deficits in plant leaf water content and are also strongly correlated with surface soil moisture. It is proposed that soil moisture deficit, which reflects the lack of sufficient moisture present in the soil for crop growth, and crop water stress, which reflects physiological stress and reduced crop vigour, should be considered separately as indicators of drought. While one factor represents the likely cause (soil moisture deficit), the other captures the effect (stress). Their combined information can provide a better index for agricultural drought monitoring and valuable insights for drought mitigation.

**Table 1:** Datasets of (a) land surface temperature (LST), (b) soil moisture, and (c) surface reflectance obtained from different sources

Name of the dataset		Resolution	Unit	Temporal range of availability	Source/reference(s)
(a) Land surface temperature (LST)					
National Aeronautics and Space Administration (NASA) Global Land Data Assimilation System Version 2 (GLDAS-2) (GLDAS_NOAH10_M V2.1)		0.25°, 1°; monthly	K	2000–2020	Beaudoing et al. (2020); Rodell et al. (2004)
Famine Early Warning Systems Network (FEWS NET) Land Data Assimilation System (FLDAS) (FLDAS_NOAH01_C_GL_M V001)		0.1°; monthly	K	2000–2020	McNally & NASA/GSFC/HSL (2018)
Moderate Resolution Imaging Spectroradiometer (MODIS) (MOD11C3 V006)		0.05°; daily	K	2000–2020	Wan et al. (2015)
Atmospheric InfraRed Sounder (AIRS)/Aqua L3 Monthly Standard Physical Retrieval (AIRS + AMSU) (AIRSX3STM 006)		1°; monthly	K	2000–2016	AIRS Science Team/J. Teixeira (2013)
AIRS/Aqua L3 Monthly Standard Physical Retrieval (AIRS-only; AIRS3STM 006)		1°; monthly	K	2000–2020	AIRS Science Team/J. Teixeira (2013)
GLDAS Variable Infiltration Capacity (VIC) Land Surface Model (GLDAS_VIC10_M)		1°; monthly	K	2000–2020	Beaudoing et al. (2020); Rodell et al. (2004)
(b) Soil moisture					
Advanced Microwave Scanning Radiometer-Earth (AMSR-E/ Aqua Level 3 (AMSRE_AVRMO 005)		1°	g/cm^3^	2002–2011	NEESPI Data Center Project (2002)
NASA-GLDAS-2 (GLDAS_NOAH10_M V2.1)		0.25°, 1°;	kg/m^2^	2000–present	Beaudoing et al. (2020); Rodell et al. (2004)
FLDAS (FLDAS_NOAH01_C_GL_M 001)		0.1°	m^3^/m^3^	2000–present	McNally & NASA/GSFC/HSL (2018)
Water Resources Information System (WRIS)-National Remote Sensing Centre (NRSC) VIC model (NRSC_VIC)		district-wise	m^3^/m^3^	June 2018–September 2020	Accessed from https://indiawris.gov.in/
(c) Surface reflectance					
MODIS/Terra Surface Reflectance (MOD09GA version 6)		500 m	–	2000–present	Vermote & Wolfe (2015); accessed from https://www.usgs.gov/
LANDSAT 7 (Landsat 7 Surface Reflectance Tier 2)		30 m	–	1999–present	Accessed from https://www.usgs.gov/

**Table 2:** List of agricultural drought indicators considered in the study

Type	Indicator	Equation	Reference
Vegetation condition	Normalized difference water index (NDWI)	$NDWI=\frac{NIR-SWIR}{NIR+SWIR}$; where $NIR, SWIR$ are respectively the near infrared and short wave infrared reflectance values at any instant	Gao (1996)
	Vegetation condition index (VCI)	$VCI=100*\frac{{NDVI}_{i}-{NDVI}_{min}}{{NDVI}_{max}-{NDVI}_{min}} ;$ where ${NDVI}_{I}$ is the normalized difference vegetation index (NDVI) value at a particular instant, and ${NDVI}_{min}, {NDVI}_{max}$ are respectively maximum and minimum NDVI values	Liu & Kogan (1996)
	Temperature condition index (TCI)	$TCI=\frac{{LST}_{max}-{LST}_{i}}{{LST}_{max}-{LST}_{min}}$; where ${LST}_{I}$ is the land surface temperature (LST) value at a particular instant, and ${LST}_{min}, {LST}_{max}$ are respectively maximum and minimum LST values	Kogan (1995)
Soil moisture stress	Soil moisture condition index (SMCI)	$SMCI=\frac{{SSM}_{i}-{SSM}_{min}}{{SSM}_{max}-{SSM}_{min}} ;$ where ${SSM}_{I}$ is the surface soil moisture value at a particular instant, and ${SSM}_{min}, {SSM}_{max}$ are respectively the minimum and maximum surface soil moisture values	Zhang & Jia (2013)
	Soil moisture deficit index (SMDI)	${SMDI}_{i}={0.5SMDI}_{i-1}+\frac{{SD}_{i}}{50}$; ${SD}_{i}=\frac{{SW}_{i}-{MSW}_{i}}{{MSW}_{i}-{MinSW}_{i}}\forall {SW}_{i}\le {MSW}_{i}$ ${SD}_{i}=\frac{{SW}_{i}-{MSW}_{i}}{{MaxSW}_{i}-{MSW}_{i}}\forall {SW}_{i}>{MSW}_{i}$ where ${SW}_{I}$ is the surface soil moisture value at a particular instant, and ${MSW}_{I},{MinSW}_{I},{MaxSW}_{i}$ are respectively the mean, minimum and maximum surface soil moisture values	Narasimhan & Srinivasan (2005)
	Soil water deficit index (SWDI)	$SWDI=\left(\frac{\theta -{\theta }_{FC}}{{\theta }_{AWC}}\right);$ where $\theta ,{\theta }_{FC}$ are respectively the volumetric soil water content value at the instant and at field capacity, while ${\theta }_{AWC}$ is the available water capacity in the soil	Martínez-Fernández et al. (2016)
Crop water stress	Shortwave infrared water stress index-1 (SIWSI-1)	$SIWSI-1=\frac{{\rho }_{5}-{\rho }_{2}}{{\rho }_{5}+{\rho }_{2}}$; where ${\rho }_{2}, {\rho }_{5}$ are respectively band 2 and band 5 of spectral reflectance. While band 2 corresponds to near infrared channel (NIR; 841–876 nm), band 5 is the shortwave infrared channel (SWIR; 1628–1652 nm)	Fensholt & Sandholt (2003)
	Shortwave infrared water stress index-2 (SIWSI-2)	$SIWSI-2=\frac{{\rho }_{6}-{\rho }_{2}}{{\rho }_{6}+{\rho }_{2}}$; where ${\rho }_{2}, {\rho }_{6}$ are respectively band 2 and band 6 of spectral reflectance. While band 2 corresponds to near infrared channel (NIR; 841–876 nm), band 6 is the shortwave infrared channel (SWIR; 1230–1250 nm)	Fensholt & Sandholt (2003)

The selection of appropriate drought indicators for agricultural drought monitoring is based on the Pearson’s correlation coefficient between district-wise yield and/or standardized yield and drought severity during the Kharif season (June to September), which is calculated at each grid point. Kharif paddy yield statistics are obtained from the annual reports of the Department of Agriculture and Farmers Empowerment, Government of Odisha. The yield data of 30 districts of Odisha from 2010 to 2017 for the Kharif paddy are used. The standardized yield ${z}_{i}$ for ${i}^{\mathrm{th}}$ year is then calculated using Eq. 1:

1 $${z}_{i}=\frac{{x}_{i}-\mu }{\sigma }$$

where ${x}_{i}$ is the observed yield of ${i}^{\mathrm{th}}$ year, and $\mu$ and $\sigma$ are the mean and standard deviation of the yield data, respectively. In addition, to calculate the meteorological drought index, the standardized precipitation index (SPI), and IMD (India Meteorological Department) gridded daily rainfall data at 0.25° spatial resolution covering India for the period from 2001 to 2019 are used (Pai et al., 2014). All drought indicators and indices are calculated at each grid location (at 0.25° and 1° resolution) in the study area for the study period from 2001 to 2019. The study area has 197 and 18 grid points at 0.25° and 1° resolutions, respectively.

### Methodology

#### Formulation of the regional combined drought index (RegCDI)

In this study, the entropy weighting method was selected for the flexible and transparent combination of input datasets into the regional combined drought index (RegCDI). Differential entropy is suitable for continuous random variables. The entropy value is therefore used in this framework to quantify the amount of useful information in each selected drought indicator. Prior to weighting, these indices are normalized to the same range, and the weights are calculated for the indices at each grid location using the methodology described below.

First, the frequency time series ${f}_{ij}$ corresponding to each normalized drought indicator ${r}_{i};r\in [\mathrm{0,1}]$ is computed as shown in Eq. 2:

2 $${f}_{ij}=\frac{{r}_{ij}}{{\sum }_{j=1}^{n}{r}_{ij}}$$

where ${r}_{ij}$ refers to the normalized value of the ${i}^{\mathrm{th}}$ drought indicator at the ${j}^{\mathrm{th}}$ time step. The entropy ${\mathrm{\rm H}}_{i}$ is then computed using Eq. 3:

3 $${\mathrm{\rm H}}_{i}=-K\sum_{j=1}^{n}{f}_{ij}\times \mathrm{ln}{f}_{ij} ;K={\left(\mathrm{ln}n\right)}^{-1}$$

and ${f}_{ij}\times \mathrm{ln}{f}_{ij}$ is defined as 0 if ${f}_{ij}=0$. The entropy-based weight ${W}_{i}$ of each of the *m* drought indicators is computed using Eq. 4:

4 $${W}_{i}=\frac{1-{\mathrm{\rm H}}_{i}}{m-{\sum }_{i=1}^{m}{\mathrm{\rm H}}_{i}};0\le {W}_{i}\le 1; \sum_{i=1}^{m}{W}_{i}=1$$

The RegCDI time series is then calculated by using Eq. 5, assuming that there are three indicators ($m=3$):

5 $${\mathrm{RegCDI}}_{j}={W}_{1}\times {r}_{1,j}+{W}_{2}\times {r}_{2,j}+{W}_{3}\times {r}_{3,j}$$

where ${W}_{1}, {W}_{2}, {W}_{3}$ are the entropy-based weights calculated at each grid location. The RegCDI classification adopted the percentile approach of the United States Drought Monitor (USDM; Svoboda et al., 2002) to define each drought category. The RegCDI classifies droughts into five categories as shown in Table 3.

**Table 3:** Drought classification using RegCDI

Category	Drought condition	Range of RegCDI
D0	Dry condition	0.20 to ≤ 0.30
D1	Moderate drought	0.10 to ≤ 0.20
D2	Severe drought	0.05 to ≤ 0.10
D3	Extreme drought	0.02 to ≤ 0.05
D4	Exceptional drought	≤ 0.02
N.B. RegCDI > 0.30 corresponds to wet condition		

#### Validation of RegCDI

The results of RegCDI are compared with the time series of some popular agricultural drought indicators, namely, the standardized annual yield of Kharif paddy rice, the input indices of RegCDI, the SPI (McKee et al., 1993) at 3-month (SPI-3) and 6-month (SPI-6) time scales, and the NDVI. The confusion matrix method, which was developed to evaluate the performance of classification algorithms, is widely used in remote sensing studies to assess accuracy (Foody, 2002), and is used to evaluate the performance of RegCDI in this study (see Supplementary Table S1). While the columns of the confusion matrix represent the classification based on the drought indicator, its rows represent the classes assigned based on the RegCDI monitoring. Two measures of agreement are derived from the confusion matrix: accuracy and the Matthews correlation coefficient (MCC; Matthews, 1975).

Accuracy is the ratio between the number of correctly classified events and the total number of drought events extracted from the confusion matrix. The accuracy of the proposed model is calculated by using Eq. 6:

6 $$\mathrm{Accuracy}=\frac{\mathrm{TN}+\mathrm{TP}}{\mathrm{TN}+\mathrm{FN}+\mathrm{TP}+\mathrm{FP}}$$

Here, true negatives (TN) and true positives (TP) indicate the number of times that there is agreement between the drought and non-drought conditions identified by the drought indicator and the RegCDI, respectively. Similarly, false positives (FP) indicate the misidentification of droughts by RegCDI, while false negatives (FN) indicate the number of incorrectly identified non-drought conditions.

The MCC calculates the Pearson product-moment correlation coefficient between actual and predicted classes or events. Its value varies between − 1 and + 1 and represents perfect misclassification and perfect classification, respectively. It is used for binary classification and is calculated by using Eq. 7:

7 $$\mathrm{MCC}=\frac{\mathrm{TP}\times \mathrm{TN}-\mathrm{FP}\times \mathrm{FN}}{\sqrt{\left(\mathrm{TP}+\mathrm{FP}\right)\times \left(\mathrm{TP}+\mathrm{FN}\right)\times \left(\mathrm{TN}+\mathrm{FP}\right)\times \left(\mathrm{TN}+\mathrm{FN}\right)}}$$

#### RegCDI for drought monitoring and early warning

**Fig. 2:**
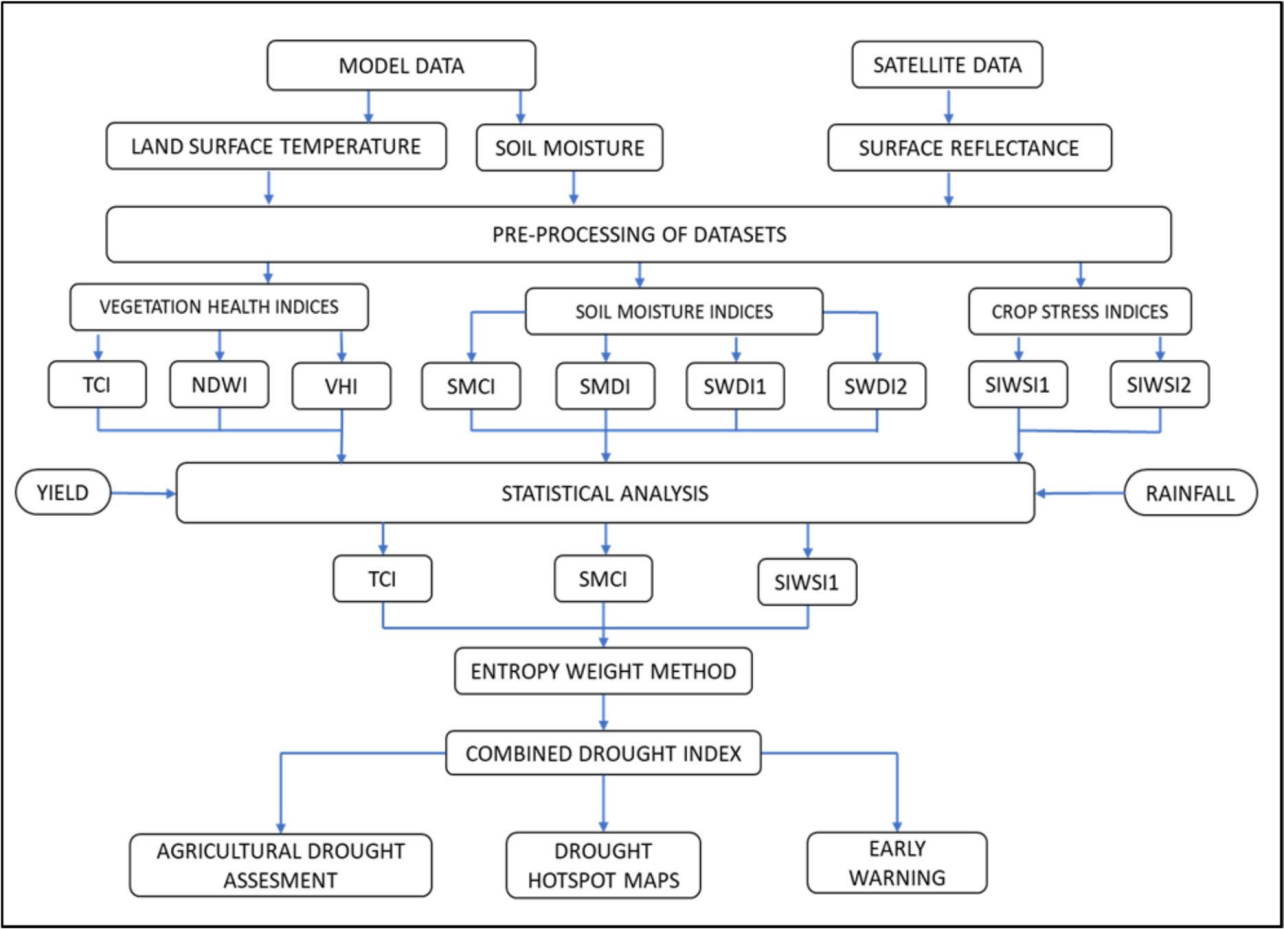
Flowchart of the regional combined drought index (RegCDI) methodology based on remote sensing products

The methodology used in this study is shown in Fig. 2. Drought monitoring with RegCDI is used for the simultaneous assessment of drought characteristics (duration and severity) as well as for the assessment of drought-affected regions and hotspots. The statistical theory of runs (Yevjevich, 1967) is used to derive drought characteristics by applying a threshold to identify the onset of drought events. Run duration is the duration of a drought event. Severity is the total magnitude of RegCDI severity in that drought duration. Drought propensity is also calculated as the number of moderate and severe RegCDI drought months and is used to identify hotspots in the study region. In this study, RegCDI drought hotspot maps are produced at both 0.25° and 1° resolutions based on the average annual drought severity, average annual drought duration, total number of drought months in the period of 2001–2019, propensity (all droughts, severe/extreme droughts), and seasonal propensity (summer, Kharif, and Rabi months).

The early drought warning capability for drought conditions is analyzed using RegCDI and its component indices. The time series of the different input indices in the RegCDI framework are evaluated for their ability to predict RegCDI drought months at different lead times: one month, two months and three months. The confusion matrix-based metrics (accuracy and MCC) are calculated to represent the accuracy and consistency of the early warning potential of the RegCDI framework at different lead times. The early warning capability of RegCDI is compared with that of the TCI by calculating the accuracy (%) and MCC between the 1- and 3-month lagged indices (RegCDI and TCI) and the drought and yield indicators (SPI-3 and SPI-6).

## Results

### Selection of drought indicators for Odisha

The soil moisture, LST, and surface reflectance datasets are used to calculate the following indices that can reflect the soil moisture level (SMCI, SMDI, and SWDI), vegetation health conditions (NDWI, VCI, and TCI), and crop stress (SIWSI-1 and SIWSI-2), respectively. Drought monitoring is carried out using these indices, and appropriate regional agricultural drought indicators for the Odisha region are selected based on the correlation of these indices with the Kharif paddy crop yield statistics (see the Appendix).

**Fig. 3:**
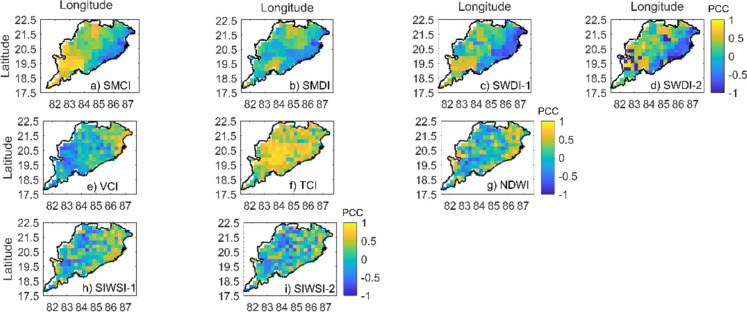
Correlations between the yield of the Kharif paddy crop and drought severity based on indices of **a** soil moisture deficit (top panel), **b** vegetation health conditions (middle panel), and **c** crop-based water stress (bottom panel) at 0.25° resolution. Yellow pixels represent improved correlations (> 0.6)

**Fig. 4:**
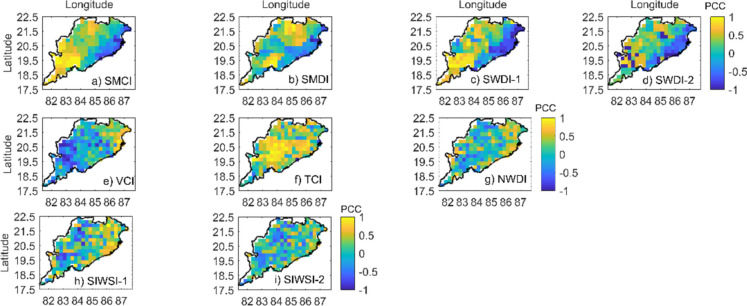
Correlations between the standardized yield of the Kharif paddy crop and drought severity based on indices of **a** soil moisture deficit (top panel), **b** vegetation health conditions (middle panel), and **c** crop-based water stress (bottom panel) at 0.25° resolution. Yellow pixels represent improved correlations (> 0.6)

**Fig. 5:**
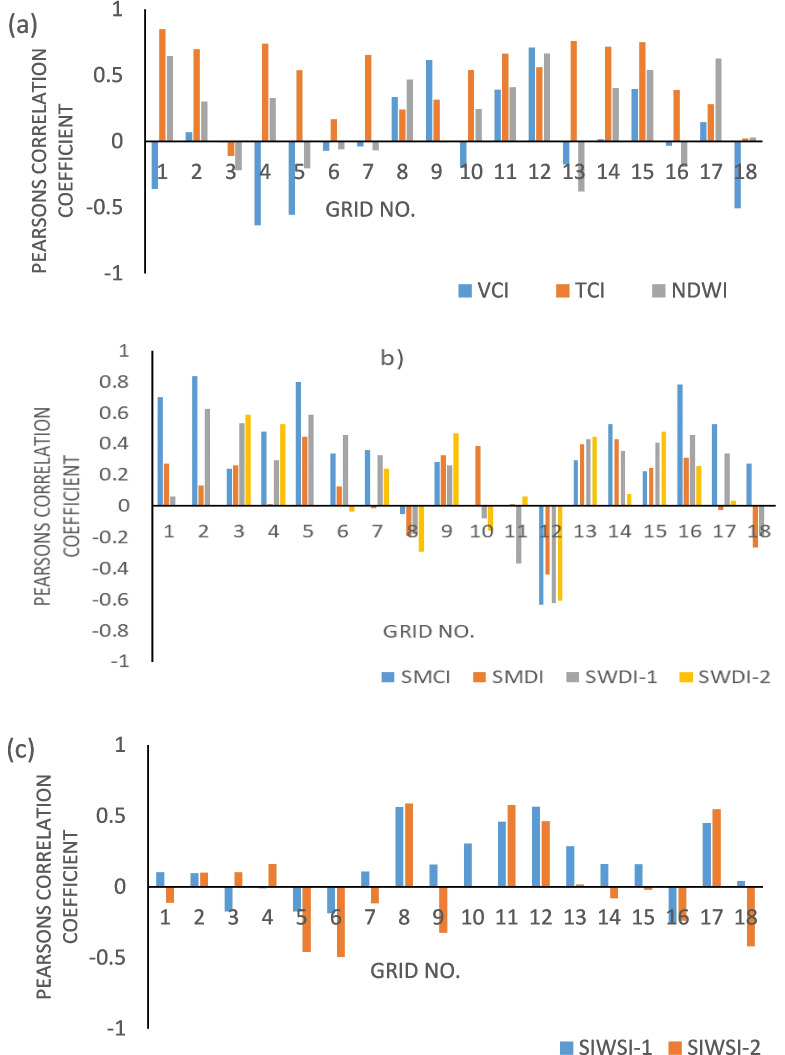
Bar plots showing the correlation between the yield of the Kharif paddy crop and drought severity based on indices of **a** soil moisture deficit, **b** vegetation health conditions, and **c** crop-based water stress at different grid points at a 1° resolution across the study area

**Fig. 6:**
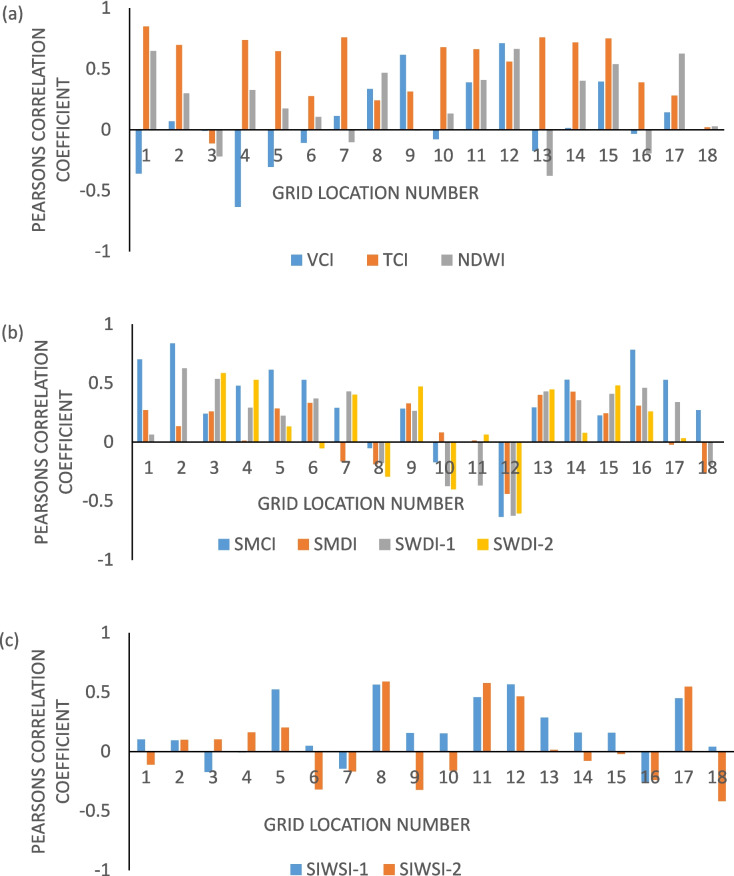
Bar plots showing the correlation between the standardized yield of the Kharif paddy crop and drought severity based on indices of **a** soil moisture deficit, **b** vegetation health conditions, and **c** crop-based water stress at different grid points at a 1° resolution across the study area

Figures 3 and 4 show Pearson’s correlation coefficient (PCC) between drought severity during the cropping season and yield at each location in Odisha, when drought monitoring using indices is carried out at 0.25° resolution. Drought severity during the Kharif season obtained from different drought indicators is compared with the standardized yield of the Kharif paddy crop by calculating the correlation coefficient at each grid point as shown in Fig. 4. Although weak correlations between drought severity and yield are observed in this study, Figs. 3 and 4 suggest that among the candidate indicators, the SMCI, TCI, and SIWSI-1 have stronger correlations with yield and standardized yield at most of the grid points at both 0.25° and 1° resolutions. The bar plots in Figs. 5 and 6 show that the TCI, SMCI, and SIWSI-1 better represent agricultural drought conditions. Previous studies by Andujar et al. (2017) also revealed that the TCI is a better indicator of drought in terms of its early onset characteristics than the VCI and VHI indices. The average Pearson’s correlation coefficient values between the regional drought indicators and yield at a 1° resolution over Odisha are presented in Table 4.

**Table 4:** The average correlation coefficients between the Kharif paddy crop yield and drought severity during the Kharif season over Odisha (at a 1° resolution)

Type of indicator	Drought indicator	Yield versus drought severity	Standardized yield versus drought severity
Vegetation condition	VCI	0.01	0.02
	TCI	0.49	0.54
	NDWI	0.20	0.20
Soil moisture deficit	SMCI	0.33	0.30
	SMDI	0.13	0.08
	SWDI-1	0.21	0.19
	SWDI-2	0.15	0.12
Crop water stress	SIWSI-1	0.15	0.15
	SIWSI-2	0.02	0.02

### Drought analysis with RegCDI

**Fig. 7:**
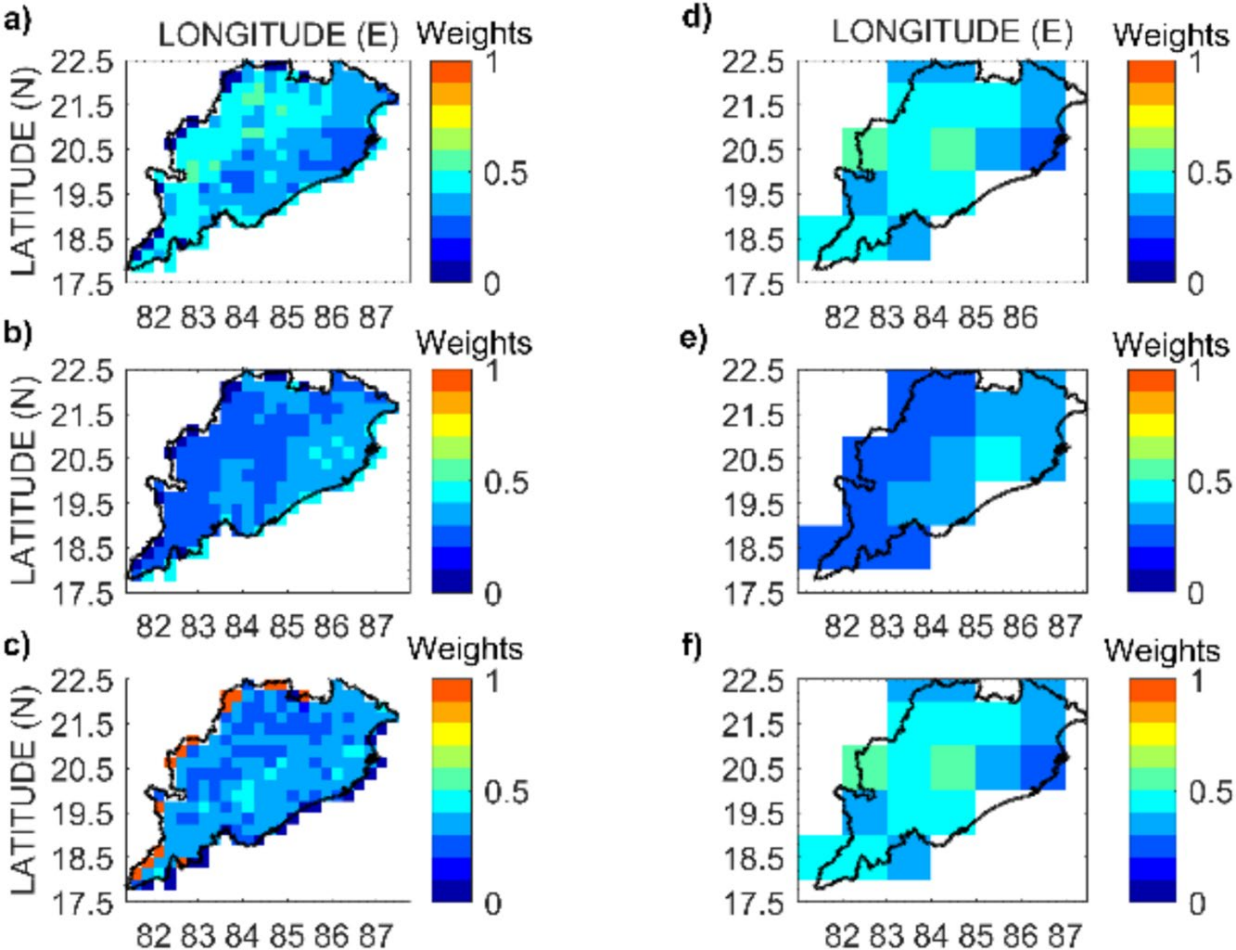
Computed weights for drought indicators used in the RegCDI framework at grid locations at 0.25° resolution (first column; plots **a**, **b**, and **c** for the SMCI, TCI and SIWSI-1, respectively), and at 1° resolution (second column; plots **d**, **e**, and **f** for the SMCI, TCI, and SIWSI-1, respectively)

**Fig. 8:**
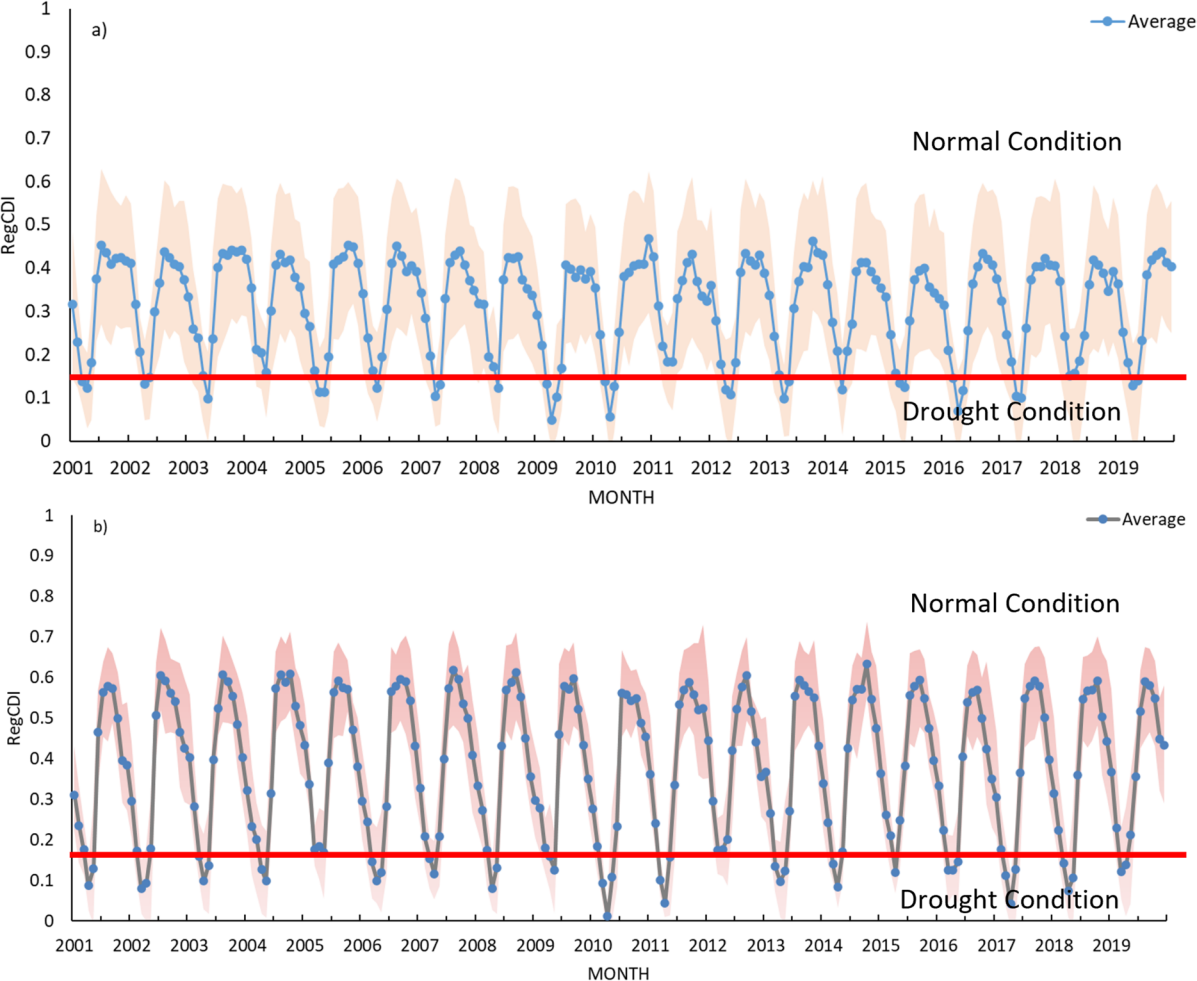
Time series of the maximum, average and minimum values of the monthly RegCDI computed across the study region at **a** 0.25° resolution and **b** 1° resolution. The RegCDI ranges between 0 and 1, representing extreme to non-drought/normal conditions, respectively

The entropy-based weights of the selected indices are calculated at each grid location separately for regional drought monitoring. After applying Shannon’s entropy method, weights are obtained for SMCI, TCI, and SIWSI-1 to estimate the RegCDI using Eq. 8:

8 $$\mathrm{RegCDI}={W}_{1}*\mathrm{SMCI}+{W}_{2}*\mathrm{TCI}+{W}_{3}*\mathrm{SIWSI}\mathrm{-1}$$

where $\mathrm{SMCI},\mathrm{ TCI},\mathrm{SIWSI}\mathrm{-1}$ are the drought indicators selected for the calculation of the new index. The spatial variation in the weights obtained for the calculation of RegCDI at 0.25° and 1° resolutions is shown in Fig. 7. Among the three constituent indicators, the SMCI and SIWSI-1 have higher weight than TCI at most of locations. The RegCDI varies between 0 and 1. Lower values indicate the most extreme drought conditions. Following the USDM percentiles for drought categorization, moderate, severe, and exceptional drought categories are defined for RegCDI, and the range of severity levels under each category is site-specific. Across all sites in the region, it is recommended that RegCDI severity values lower than 0.12 at 0.25° resolution (similarly, 0.17 for 1° resolution RegCDI) should be used as the threshold for drought conditions for confusion matrix-based analysis. The time series plots of the maximum, minimum, and mean values of RegCDI calculated over all sites in the study area from 2001 to 2019 are shown in Fig. 8.

#### Performance validation of RegCDI

The confusion matrix is used to calculate metrics such as accuracy and MCC for the assessment of drought conditions as represented by RegCDI and other popular indices and yields. Table 5 lists the metrics based on the confusion matrix method. The minimum values of the RegCDI and the input indices (SMCI, TCI, and SIWSI-1) during the Kharif season are categorized into either drought or non-drought conditions according to the classification against the standardized yield. The MCC is relatively low (for both resolutions), indicating that the categorization is not consistent. The predicted value, i.e., the minimum severity of the indices in the Kharif season, is not much correlated with the actual value, i.e., the standardized yield of Kharif paddy rice (Table 5). On the other hand, the accuracy and the MCC value are higher (in both cases of analysis) when the severity values during the critical period are taken as the predicted value against the actual values of the standardized yield of Kharif paddy (Table 6) which confirms the good agreement between the yield and the RegCDI (and its input indices). Again, the accuracy of categorizing the RegCDI into drought and non-drought conditions is checked by using a confusion matrix with respect to the input indices (SMCI, TCI, and SIWSI-1) and other indices (SPI-3, SPI-6, and NDVI). The analysis revealed that the accuracy improved with increasing MCC, as shown in Table 7, from which it can be concluded that RegCDI has a better correlation with the climatic indices and can be used to accurately classify drought and non-drought conditions. However, when RegCDI categorizes the conditions into five categories, ranging from no drought, to mild drought, severe drought, extreme drought, and exceptional drought conditions, the accuracy decreases in comparison to binary classification (Table 7) in both cases of the study. The validation of RegCDI using the standardized yield versus drought severity during the crop growth stage month in the Kharif season (at 0.25° and 1°) showed its ability to detect local drought during the critical period of the agricultural season. However, validation studies are often not easily feasible as local scale crop yield data and continuous drought monitoring data are not available as reliable records for different regions, especially because they are evaluated at regional rather than local scales.

**Table 5:** Measures of agreement: accuracy (%) and Matthews correlation coefficient (MCC) extracted from confusion matrices between standardized yield and maximum drought severity in the Kharif season computed at (a) 0.25° and (b) 1° resolution using different indicators and RegCDI

	Index	Accuracy (%)	MCC
(a) Standardized yield versus drought monitoring at 0.25° resolution			
1	RegCDI	48.01	− 0.03
2	SMCI	49.81	–
3	TCI	49.85	–
4	SIWSI-1	48.74	− 0.05
(b) Standardized yield versus drought monitoring at 1° resolution			
1	RegCDI	48.96	− 0.06
2	SMCI	51.04	–
3	TCI	51.04	–
4	SIWSI-1	48.96	− 0.02

**Table 6:** Measures of agreement: Accuracy (%) and Matthews correlation coefficient (MCC) extracted from confusion matrix between standardized yield and drought severity in the critical crop growth stage month in the Kharif season computed at (a) 0.25° and (b) 1° resolution using different indicators and RegCDI

	Index	Accuracy (%)	MCC
(a) Standardized yield versus critical period drought monitoring at 0.25° resolution			
1	RegCDI	51.04	0.02
2	SMCI	49.08	–
3	TCI	55.59	0.11
4	SIWSI-1	47.42	− 0.06
(b) Standardized yield versus critical period drought monitoring at 1° resolution			
1	RegCDI	58.04	0.13
2	SMCI	47.92	–
3	TCI	54.17	0.10
4	SIWSI-1	47.22	− 0.04

**Table 7:** Measures of agreement accuracy (%) and Matthews correlation coefficient (MCC) extracted from confusion matrices between drought classification of RegCDI and different drought indices computed at (a) 0.25° and (b) 1° resolution

			Binary classification (no drought, drought)			Multi-class (no drought, mild drought, severe, extreme, and exceptional drought)
		Index	Accuracy (%)		MCC	Accuracy (%)
(a) RegCDI versus drought indices at 0.25° resolution						
1		SMCI	87.53		0.69	82.74
2		TCI	91.21		0.78	84.25
3		SIWSI-1	90.04		0.76	87.19
4		SPI-3	80.89		0.49	74.04
5		SPI-6	80.40		0.49	74.90
6		NDVI	79.98		0.48	59.20
(b) RegCDI versus drought indices at 1° resolution						
1		SMCI	67.86		0.47	60.21
2		TCI	79.75		0.54	67.37
3		SIWSI-1	80.04		0.49	76.54
4		SPI-3	50.76		0.01	44.40
5		SPI-6	50.24		-0.01	43.96
6		NDVI	49.68		-0.24	40.04

#### Regional drought hotspot analysis

**Fig. 9:**
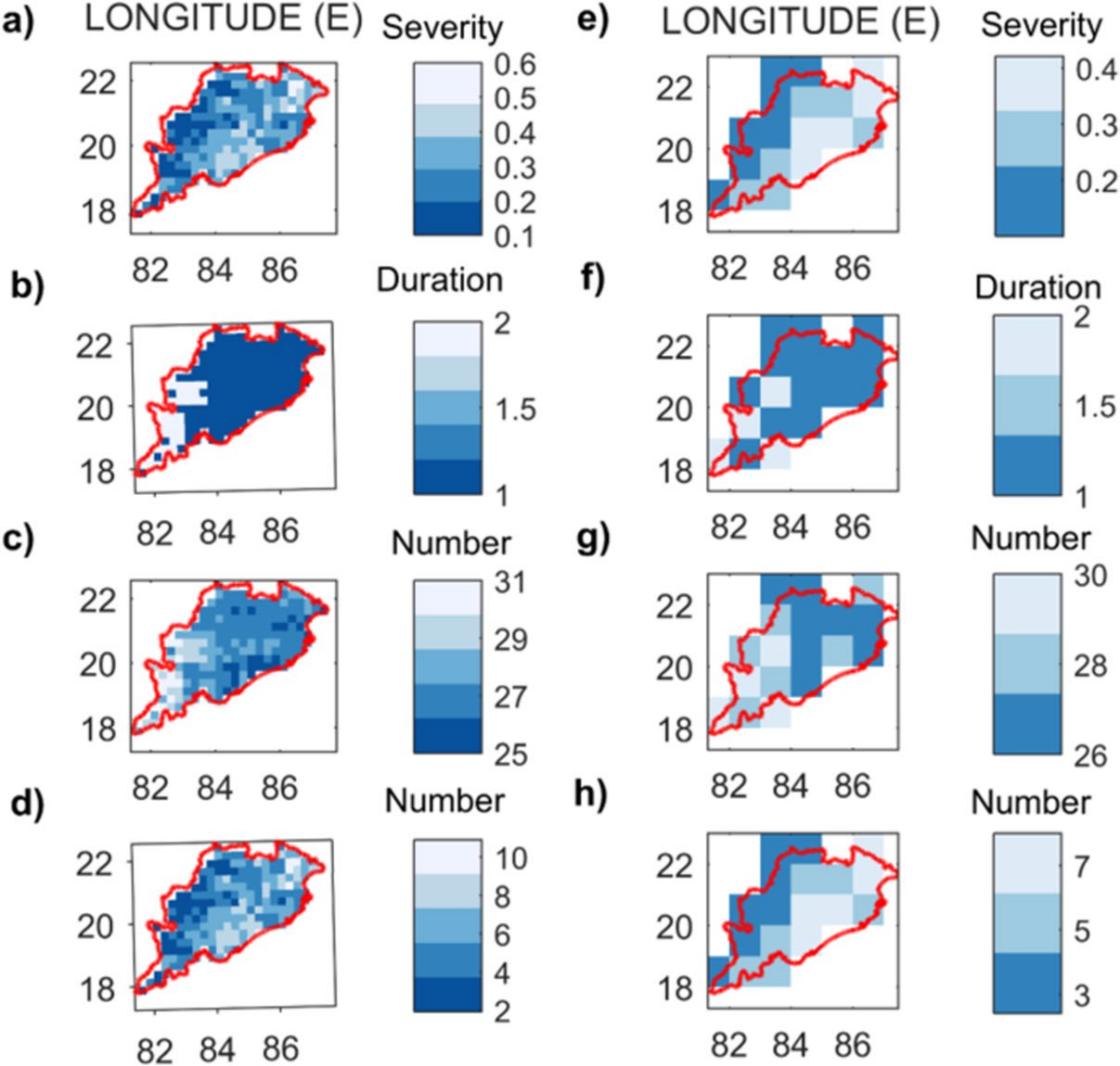
Drought hotspot maps of Odisha based on **a** average annual drought severity, **b** average annual drought duration, **c** total number of drought months, and **d** total number of severe to exceptional category drought months computed using RegCDI at 0.25° resolution (left column panels) and 1° resolution (right column panels) during 2001–2019. Locations with lighter pixels have increased drought risk as well as higher magnitudes of drought severity and duration

Drought hotspot analysis and agricultural drought monitoring in Odisha are based on the drought characteristics of the developed combined index. In this regard, the average annual drought severity, annual drought duration, number of drought months showing mild to extreme conditions, and only severe to extreme drought conditions at each grid point during the period 2001–2019 are determined and are shown in plots (a) to (d), respectively, of Fig. 9. Hotspot analysis revealed that the western and southwestern parts of Odisha have the lowest values of mean annual severity (i.e., the lower the severity value is, the more severe the drought conditions are), the higher mean annual duration of drought, and a greater number of months classified as mild to extreme drought.

**Fig. 10:**
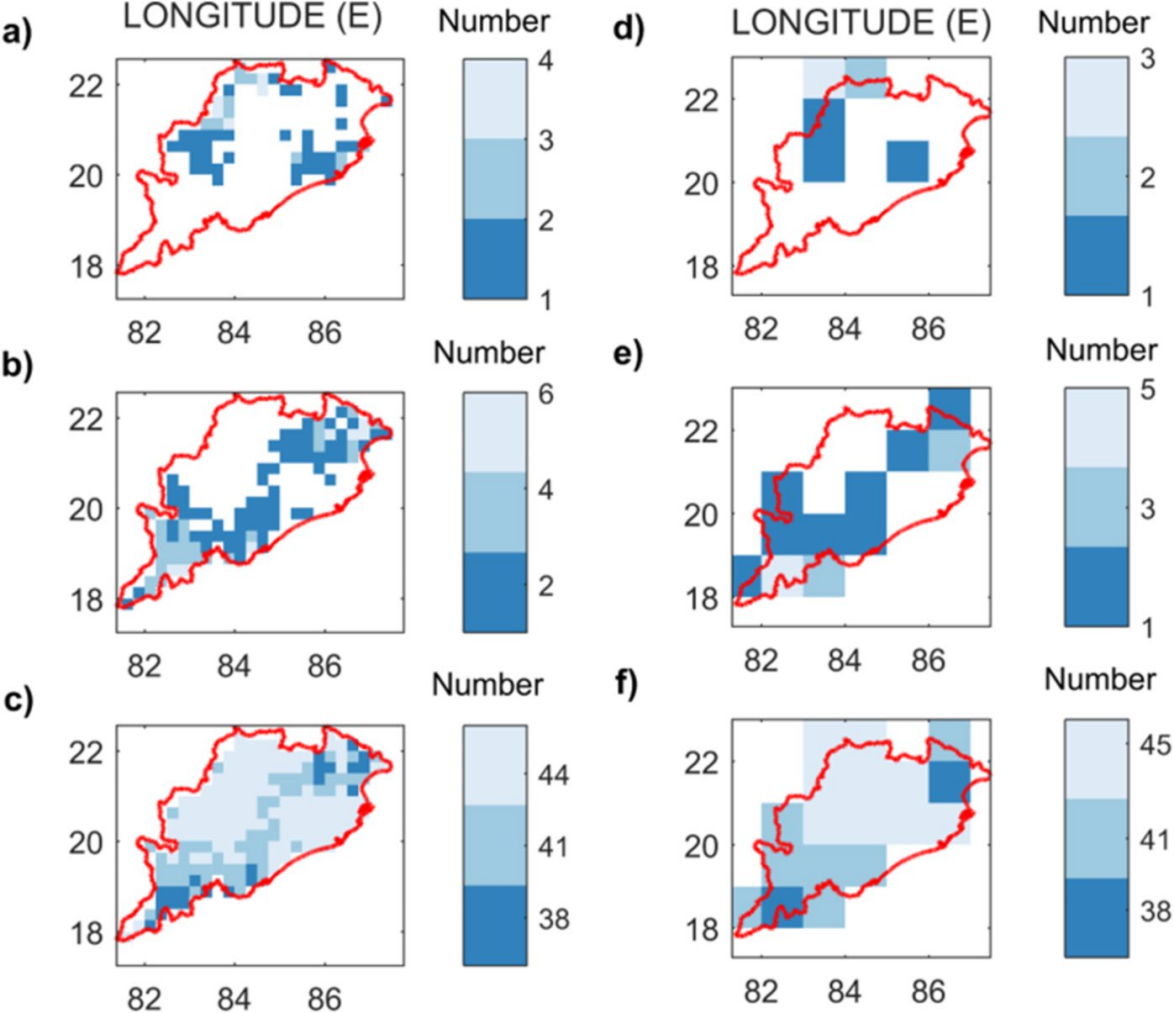
Drought hotspot maps of Odisha based on the total number of extreme category drought months during crop seasons: **a** Kharif, **b** Rabi, and **c** Summer computed using RegCDI at 0.25° resolution (left column panels) and 1° resolution (right column panels) during 2001–2019. Locations with lighter pixels have increased drought risk as well as higher magnitudes of drought severity and duration, and the summer season is the most vulnerable to droughts in this region

The study area is cultivated throughout the year during the Kharif, Rabi, and summer cropping seasons. While Kharif crops (paddy) are the most common and account for the majority of regional agricultural productivity, Rabi (winter) and summer crops are becoming increasingly popular for increasing annual crop production. The Kharif season falls during the rainy months of the year, so agricultural droughts are predicted to be minimal. The severe to extreme drought conditions of the three cropping seasons are contrasted to determine the temporal drought vulnerability of the region. Figure 10a–c shows the drought hotspot maps generated using the RegCDI for the Kharif, Rabi, and summer seasons, respectively (at 0.25° and 1° resolution), showing the number of severe events at each location during the period 2001–2019. These results indicate that rabi crops are susceptible to droughts, with summer crops being the most affected as these are the periods with the lowest rainfall. Overall, droughts are less frequent during the Kharif season.

### Analysis of the drought early warning potential of RegCDI

**Fig. 11:**
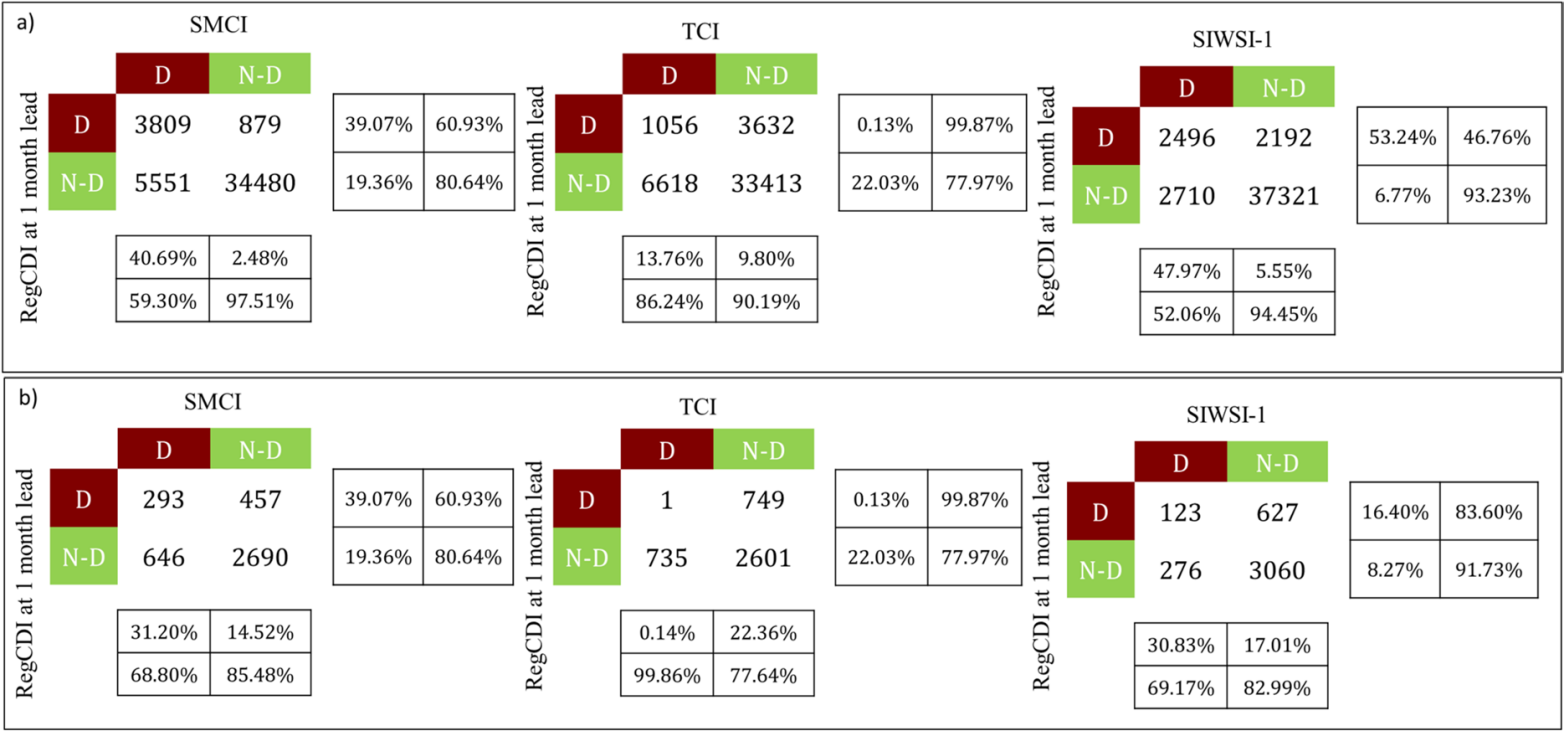
Confusion matrices between RegCDI at a one-month lead time and the drought indicators SMCI, TCI, and SIWSI-1, performed at **a** 0.25° resolution and **b** 1° resolution. Here, drought early warning performance analysis is performed for binary classification into drought and non-drought categories

The drought early warning capacity and accuracy of the RegCDI framework are assessed via confusion matrix analysis between the RegCDI and the 1-month (Fig. 11), 2-month, and 3-month lagged SMCI, TCI, and SIWSI-1. The confusion matrix shows the number of severe drought months and non-drought months according to the RegCDI (by using a threshold value of 0.12 and 0.17, which is the average of the severity of severe drought conditions of all the pixels at 0.25° and 1°, respectively) corresponding to the lagged SMCI, TCI, and SIWSI-1. The degree of agreement between them at both resolutions is shown in Table 8. SMCI and SIWSI-1 are found to be suitable for predicting RegCDI droughts at 1-month and 2-month lead times, while TCI has the lowest potential to indicate RegCDI droughts at all lead times. This could be due to the role of the soil moisture deficit in the onset of drought, which is better represented by the SMCI and SIWSI-1 than by the TCI. Accuracy and MCC values at 1-month lag are higher than those at 2- and 3-month lag for all the indices at both spatial resolutions, indicating the capability of short-term drought forecasting using these two indices. Additionally, the performance of RegCDI is better than that of TCI at 2- and 3-month lead times (Table 9) when both are compared with that of SPI-3 and SPI6. Therefore, early warning can be provided one month before the onset of severe drought conditions by using the SMCI and SIWSI-1 in this study region.

**Table 8:** Measures of agreement related to drought early warning capability of RegCDI at (a) 0.25° resolution and (b) 1° resolution, respectively, extracted from confusion matrices of binary class drought classification. Accuracy (%) and Matthews correlation coefficient (MCC) are estimated using RegCDI at 1- to 3-month lead times and drought indicators (SMCI, TCI, and SIWSI-1)

RegCDI lead time	1 month		2 months		3 months	
Indicator	Accuracy (%)	MCC	Accuracy (%)	MCC	Accuracy (%)	MCC
(a) Drought early warning using RegCDI at 0.25° resolution						
SMCI	85.62	0.51	82.97	0.40	77.45	0.17
TCI	77.07	0.05	72.36	− 0.15	72.58	− 0.15
SIWSI-1	89.04	0.44	86.68	0.32	83.52	0.15
(b) Drought early warning using RegCDI at 1° resolution						
SMCI	87.4	0.62	82.23	0.46	73.01	0.18
TCI	71.46	0.05	63.80	− 0.21	63.68	− 0.22
SIWSI-1	85.90	0.47	81.96	0.29	77.90	0.11

**Table 9:** Measures of agreement related to drought early warning capability of (a) RegCDI and (b) TCI at 0.25° resolution and 1° resolution, extracted from confusion matrices of binary class drought classification. Accuracy (%) and Matthews correlation coefficient (MCC) are estimated using RegCDI and TCI at 1- to 3-month lag times and drought and yield indicators (SPI-3, SPI-6)

Indicator	Accuracy (%)	MCC	Accuracy (%)	MCC	Accuracy (%)	MCC
(a) Drought early warning using RegCDI						
RegCDI lead time	1 month		2 months		3 months	
SPI-3 (0.25° resolution)	51.41	0.03	52.06	0.05	51.41	0.03
SPI-6 (0.25° resolution)	51.12	0.02	51.60	0.04	51.29	0.03
SPI-3 (1° resolution)	52.10	0.04	52.77	0.05	52.25	0.04
SPI-6 (1° resolution)	51.10	0.01	51.71	0.03	52.13	0.04
(b) Drought early warning using TCI						
TCI lead time	1 month		2 months		3 months	
SPI-3 (0.25° resolution)	52.46	0.05	50.92	0.02	50.83	0
SPI-6 (0.25° resolution)	51.94	0.04	0.70	− 0.09	0.70	− 0.09
SPI-3 (1° resolution)	52.84	0.06	51.30	0.01	51.37	0.01
SPI-6 (1° resolution)	52.08	0.04	0.54	− 0.12	0.54	− 0.12

## Discussion

### Need and merits of the RegCDI framework

The formulation of RegCDI using locally relevant selected constituent indicator variables for the Odisha region, such as soil moisture, LST and surface reflectance, is in line with numerous recent studies reported in the literature (Bayissa et al., 2018; Chattopadhyay et al., 2020; Das et al., 2020; Dilip et al., 2023; Han & Singh, 2023; Kulkarni et al., 2020; Mullapudi et al., 2023). The merits of a Combined Drought Indicator (CDI) framework for agricultural drought monitoring are demonstrated in the performance validation phase, as well as in its capabilities for early drought warning. It is important to understand the basis of the drought indicator components and whether they are well suited to the region under study. The RegCDI framework is flexible for choosing other indicators than in this case study. The entropy method allows their weighing according to their informational content. Such data-based approach depends on the availability and characteristics of the input data and may lead to different results when different data sources are used.

In a previous work by Sepulcre-Canto et al. (2012), a CDI was developed for the European region, with the standardized precipitation index (SPI), soil moisture anomaly and fraction of Absorbed Photosynthetically Active Radiation (fAPAR) anomaly as constituents. The choice of these constituents was primarily made to address the “watch,” “warning,” and “alert,” respectively, which also served as early warning system. Similarly, Chattopadhyay et al. (2020) developed a CDI that combines meteorological, soil moisture and remotely sensed information. In their study, while soil moisture, rainfall, and the soil water deficit index had higher weighting components, other aspects such as the crop moisture index, evaporative stress index, normalized differential vegetation index, vegetation condition index, temperature condition index, and vegetation health index had lower weighting in the formulation of the index. The regional CDI proposed by Kulkarni et al. (2020) used datasets for the LST, SPI, NDVI, and SM for the Marathwada (CDI_M) region of India. Compared to these CDIs, RegCDI reduced the number of component indicators retaining only those that are well suited for assessing regional drought conditions. An assessment of the suitability of the selected indicator variables is lacking in most other studies. A recent study by Dilip et al. (2023) developed their own CDI as a weighted sum of the SPI, the SMI and the area sown. Their hypothesis for selecting crop area as an indicator was that, during periods of rainfall deficit, which could lead to agricultural drought, one would observe delayed sowing practices that eventually reduce the extent of the cropped area. In their study, they used remote sensing data to measure the area sown with crops.

The proposed framework provides a flexible model for drought monitoring, as evidenced by the adaptability of the model at different spatial resolutions, driven by the availability of input data. The assignment of weights using the scientific basis provided by the entropy weighting method shows that the main driver of drought is indeed soil moisture deficiency and the resulting crop stress, while temperature effects have a relatively minor contribution. In addition, the weights assigned to the indices (Fig. 7) could vary spatially, and RegCDI could capture diverse patterns of drought conditions spatially, as shown in Fig. 9 (within Odisha).

The analysis of drought-prone regions across Odisha at 0.25° and 1° spatial resolutions using RegCDI provided interesting insights. The hotspot analysis based on the severity of the RegCDI indicated that the western and southwestern parts of Odisha are severely affected by drought. The duration and severity of drought events are maximum in the southwestern parts, mainly in the Kalahandi, Kandhamal, and Balangir districts of Odisha (see Supplementary Fig. S1), and are therefore considered drought hotspots (Swain & Swain, 2011). The identified hotspots (western and southwestern regions of Odisha) can be attributed to decreasing trend in rainfall and increasing trend in temperature and evaporation between 1901 and 2019 (Saha et al., 2021). These areas need drought adaptation and mitigation support in the form of additional irrigation facilities and sustainable farming practices to reduce crop losses. The seasonal assessment of drought events also suggested that the Odisha region has experienced two to more than a dozen times more moderate to severe droughts during the Rabi and summer seasons, respectively, than during the Kharif season. During the non-rainy seasons, there is a need to consider sustainable irrigation practices in the area, as well as the choice of drought-tolerant crop varieties. Drought-tolerant crop varieties, rather than paddy cultivation, are better suited for these seasons to reduce overall crop water stress in the region.

The ability of RegCDI to provide early warning of agricultural drought is undoubtedly an important contribution of the present study. In particular, an integrated framework such as RegCDI is found to outperform individual indices such as the TCI, SMCI, and SIWSI-1 for drought early warning with up to a 1-month lead time. Most of the indices operating at weekly to monthly time scales are limited by early warning capabilities at the corresponding time scales (Shukla et al., 2020). Indeed, the CDI of Sepulcre-Canto et al. (2012) relied on the SPI component of the framework for drought “watch,” the soil moisture anomaly for drought “warning” and the fAPAR anomaly for “alert.” However, the study lacked a quantitative assessment of the early warning model. Previous studies, such as that by Shukla et al. (2020), have shown that early warning performance beyond three months is quite low (correlations below 0.3), whereas correlations in the range of 0.40–0.50 are obtained for early warnings with a one-month lead time.

### Insights from the validation of drought monitoring

During the validation of RegCDI, the correlation between the standardized yield and drought severity (at both 0.25° and 1° resolutions) during the crop growth stage in the Kharif season indicated its ability to detect local agricultural droughts. Similar findings have been reported in the drought literature, although a quantitative assessment of validation results using statistical measures and drought onset characteristics is still lacking. This is particularly true in the Indian context due to the paucity of continuous long-term yield data and has limited the validity of the present study.

Few studies have examined the correlation between the agricultural drought index and crop yield to assess the performance of the index. In their study, Andujar et al. (2017) reported a low correlation of vegetation indices with the meteorological drought indicator SPEI at different time scales. Patel et al. (2012) obtained significant but weak coefficient of determination values (*R*^2^; 0.20–0.35) between VTCI drought duration and yields of food grains and oilseeds. Dutta et al. (2013) calculated the NDVI for different 14-day intervals, and found that the NDVI of the first 14-day period of a growing season correlated well with the yield of the season. Patel & Yadav (2015) estimated the correlation between food grain yield anomalies and the VCI and found that the correlations were in the range of 0.28–0.81, varying with the time period of the year. Zhuo et al. (2016) reported that positive correlations between indices such as the VCI, VHI and TCI and the standardized winter wheat yield reached 0.40 during the flowering-filling stage. Satellite-based vegetation drought indices are more strongly correlated with yield and yield anomalies than the meteorological drought indices (García-León et al., 2019). Looking at previous studies on combined indices, Sepulcre-Canto et al. (2012) compared annual crop production in terms of cereal yields during watch, warning or alert periods to determine whether drought monitoring was effective for the European region. At a few stations, the authors found that the decline in crop yields corresponded well with the drought conditions, indicating the suitability of their CDI. Kulkarni et al. (2020) calculated the correlation between droughts and yields of different crops over an 18-year period. The CDI_M of Kulkarni et al. (2020) reported very good correlation values (0.60) with crop yields in both the Kharif and Rabi seasons, and even higher agreement of yields with droughts in drier years. Using their proposed CDI, drought monitoring at a 0.25° spatial resolution by Chattopadhyay et al. (2020) could not provide significant correlations in all study regions equally when droughts were compared with district-wise crop yields. Dilip et al. (2023) reported a significantly high correlation of CDI-based drought monitoring with paddy yield and a good correlation with yields of other crops such as cotton in their study area.

The challenging question of validating a drought monitoring framework has been addressed by many researchers through visual comparisons of drought severity maps. Authors often select specific drought years and/or months to investigate how well their proposed index captures drought extent, severity and propagation in time and space compared to standard drought indices. For example, when Das et al. (2020) proposed a novel multivariate phenology-based agricultural drought index (MADI), they compared maps of drought categorization during a few drought periods to assess the performance of the MADI. However, validation studies are often challenging because local scale crop yield data and continuous drought monitoring data are not available as reliable records for all regions equally, especially because they are assessed at regional rather than local scales.

### Future directions

A close examination of the results of different regional drought monitoring studies reported in the literature highlights the challenges faced in developing an appropriate generalized framework, as well as the research gaps that suggest the future scope of research in agricultural drought monitoring. The usefulness and scope of popular methods such as PCA and copulas for integrating numerous remotely sensed drought-related variables into the CDI framework need to be investigated through case studies. There are numerous recent satellite datasets with finer spatial and temporal resolutions (Bauer-Marschallinger et al., 2018; Dilip et al., 2023) that are easily accessible to the public and can provide continuous drought monitoring and help in agricultural water resource management in water-stressed areas. The agricultural drought hotspots identified by regionally-based indices and their early warning alerts can facilitate better drought mitigation by various policy makers and stakeholders. As a precautionary measure, farmers in the identified hotspot regions can adopt low water-demand crops or drought-resistant varieties and adjust sowing dates to minimize crop losses due to drought events (Dilip et al., 2023). Certainly, such an integrated target area identification and subsequent policy formulation strategy could effectively combat the adverse effects of drought and promote timely irrigation application in the target areas.

## Conclusions

The proposed RegCDI framework provides useful insights for drought management, and can help minimize yield losses and the economic impacts of agricultural droughts on different stakeholders. The choice of the agricultural parameters that primarily affect agricultural productivity, as well as the variables and indices that best represent these parameters, is important. While this study used readily available data sources and easy-to-use drought indicators for vegetation conditions, soil moisture levels, and crop water stress, the proposed framework needs to be tested for the particular study area to determine the choice of indicators.

The performance of the index is measured via correlation analysis with the paddy yield during the Kharif season. Confusion matrix analysis is also used to validate the performance of RegCDI against yield, input indicators, and other popular indices. The findings emphasize the need for comprehensive ground truthing of the index with observed yield and crop growth indicators for its best practical application. The drought characteristics extracted from RegCDI are also able to locate drought hotspots in the study area based on long-term drought severity and where severe and prolonged drought conditions prevailed. The drought hotspots in the region with seasonal drought patterns could plan for more efficient irrigation, drought resistant varieties, and sustainable cropping systems in the Rabi and summer seasons to reduce agricultural losses and their impact on people’s socio-economic status. The early warning potential of the index and its component indicators is assessed using confusion matrix analysis for lead times up to 3 months. The constituent indices, SMCI and SIWSI-1, can be efficiently used for early warning of drought using RegCDI with a lead time of one to two months.

Overall, agricultural drought monitoring using RegCDI is more beneficial because it represents different indicators of drought rather than a single indicator of drought. As the drought validation analysis suggested variable correlations between drought monitoring and crop yield depending on the growth stage of the crop, the framework needs to be thoroughly validated for all crop seasons and growth stages by comparing it with popular drought indices (SPEI, etc.), which is a future scope of study. Despite the coarse resolution, the framework can be adopted and modified to suit drought studies at local-to-regional scales by relying on high resolution RS-based datasets. However, validation is not quite feasible in our opinion, except when high-resolution plot-scale yield data are made available for public use by governing bodies and authorities. In this way, the proposed drought monitoring and early warning framework will become more helpful to end users.

## Electronic supplementary material

Below is the link to the electronic supplementary material.

Supplementary file1 (DOCX 269 KB)

## Data Availability

The precipitation dataset is available at https://www.imdpune.gov.in/cmpg/Griddata/Rainfall_25_Bin.html. The MODIS surface reflectance data (MOD09GA V006) and MODIS land surface temperature (LST) data (MOD11C3 V006) are available at 10.5067/MODIS/MOD09GA.006 and https://appeears.earthdatacloud.nasa.gov, respectively. The land surface temperature (LST) data AIRSX3STM 006 and AIRS3STM 006 are available at 10.5067/Aqua/AIRS/DATA319. The GLDAS soil moisture and LST data (GLDAS_NOAH10_M V2.1, GLDAS_VIC10_M) are available at https://disc.gsfc.nasa.gov/datacollection/GLDAS_NOAH10_M_EP_2.1.html. The FLDAS data (LST and soil moisture FLDAS_NOAH01_C_GL_M V001) and AMSRE data (AMSRE_AVRMO 005) are available at https://disc.gsfc.nasa.gov/datasets. Soil moisture data from the NRSC_VIC model are available at https://indiawris.gov.in/. The district-wise annual yield of Odisha is available at https://agri.odisha.gov.in.

## Appendix. Data selection and pre-processing

The datasets listed in Table 1 are considered to compute indicators representing vegetation health conditions, soil moisture levels, and crop water stress, which are then integrated to develop a new regional combined drought index. To select the best datasets of LST and soil moisture, the GLDAS_VIC model data and the NRSC_VIC model data, respectively, are adopted as proxy observational reference data. Bias correction is performed to improve the match of the available LST, soil moisture, and surface reflectance datasets with the chosen reference data. The quantile mapping technique (Panofsky & Brier, 1968) is used, which transforms the cumulative distribution function (CDF) of the simulated variable of interest onto the observed CDF to remove the bias present between model simulations and observations. Quantile mapping has been applied in several climate modeling studies (Piani et al., 2010). The gamma distribution is used as the continuous probability distribution function for transformation of the CDF.

The performance of the different datasets is evaluated using statistical analysis and the metrics listed in Supplementary Table S2. The chosen datasets are prepared at monthly time step and two spatial resolutions: 0.25° and 1° using regridding and bilinear interpolation techniques for agricultural drought monitoring over the Odisha region. The input datasets for RegCDI available from different sources, including remote sensing, are compared with available ground-truth data or proxies of observational data to select those with the least bias. Accordingly, GLDAS datasets of LST (Supplementary Table S3a) and bias-corrected soil moisture (Supplementary Table S3b) with the highest *R*^2^ and least average bias and RMSE values, as well as longer period of data, are selected. Bias correction significantly improved the quality of input soil moisture data, for example, comparison plots of the bias-corrected soil moisture data from different models and the NRSC_VIC model data (ground truth data) at a grid location (21.5° N, 86.5° E) at both 0.25° and 1° spatial resolutions are shown in Supplementary Figure S2. In general, the correction of daily soil moisture values is limited, as seen in the plot. However, the drought analysis is at monthly time steps, which decreases the discrepancies. In the case of surface reflectance, the MODIS MOD09GA (with 500-m grid resolution) dataset is chosen over the LANDSAT-7 (30-m resolution) dataset because the regridding error (to 0.25° and 1° resolution) is lower in MOD09GA than in LANDSAT-7. The performance evaluation of the input data sources and the selected datasets for use in the study are summarized in Supplementary Tables S3 and S4, respectively. These datasets are used for drought monitoring at monthly time steps in this study.
