# Supplementary material for: Agricultural drought monitoring and early warning at the regional scale using a remote sensing-based combined index
Source: Environ Monit Assess. 2024 Oct 30;196(11):1132. doi: 10.1007/s10661-024-13265-y (PMC11525277; doi:10.1007/s10661-024-13265-y)
Supplement: Supplementary file 1 — Supplementary file1 (DOCX 269 KB) [file 10661_2024_13265_MOESM1_ESM.docx]

**LIST OF SUPPLEMENTARY TABLES**

**Table S1.** Confusion matrix adopted in the study for assessment of performance of RegCDI

| Drought indicator class🡪  RegCDI class | Non-drought (positive) | Drought (negative) |
| --- | --- | --- |
| Non-drought (positive) | True Positive (TP) | False Positive (FP) |
| Drought (negative) | False Negative (FN) | True Negative (TN) |

**Table S2.** Statistical metrics used for selection of the dataset representing different drought variables. Here, $M_{n}$ and $O_{n}$are respectively the $n^{th}$ modeled and observed value in the dataset which has sample size $N$

| Sl. No. | Statistic | Equation |
| --- | --- | --- |
| 1 | Pearson correlation coefficient (PCC) | $\frac{N*\sum M_{n}O_{n}-\sum M_{n}\sum O_{n}}{\sqrt{\left( N*\sum{M_{n}}^{2}-({\sum M_{n})}^{2} \right)\left( N*\sum{O_{n}}^{2}-({\sum O_{n})}^{2} \right)}}$ |
| 2 | Average bias error (Bias) | $\frac{1}{N}\sum\left( M_{n}-O_{n} \right)$ |
| 3 | Root-mean-square error (RMSE) | $\sqrt{{\sum\left( M_{n}-O_{n} \right)^{2}}/N}$ |

**Table S3.** Performance metrics-based evaluation of (a) land surface temperature (LST) datasets versus GLDAS_VIC model dataset and (b) soil moisture datasets versus NRSC VIC model dataset. Average bias, coefficient of determination (R^2^) and root mean square error (RMSE) are used for evaluating the performance, and in the absence of field observations, the GLDAS_VIC and NRSC VIC datasets are adopted for ground-truthing.

| (a) LST | | | | |
| --- | --- | --- | --- | --- |
|  | MOD11C3 V006 versus GLDAS_VIC | FLDAS_NOAH01_C_GL_MV001 versus  GLDAS_VIC | | GLDAS NOAH10_M V2.1 versus  GLDAS_VIC |
| Average Bias | -3.52 | 1.40 | | 0.57 |
| *R*^2^ | 0.42 | 0.97 | | 0.99 |
| RMSE | 5.74 | 1.87 | | 0.98 |
|  | | | | |
| (b) Soil moisture | | | | |
|  | Bias corrected GLDAS_NOAH10_M 2.1 versus  NRSC VIC | | Bias corrected FLDAS_NOAH01_C_GL_M 001 versus  NRSC VIC | |
| Average Bias | -0.02 | | -0.02 | |
| *R*^2^ | 0.70 | | 0.49 | |
| RMSE | 0.07 | | 0.08 | |

**Table S4.** Datasets selected in the study for agricultural drought monitoring

| **Drought indicator** | **Source** | **Spatial resolution** | **Time period** |
| --- | --- | --- | --- |
| **Precipitation** | IMD | 0.25°, 1° | 2001-2019 |
| **Soil moisture** | GLDAS_NOAH10_M V2.1 | 0.25°, 1° | 2001-2019 |
| **LST** | GLDAS_NOAH10_M V2.1 | 0.25°, 1° | 2001-2019 |
| **Surface reflectance** | MOD09GA | 500 m | 2001-2019 |

**Table S5.** Table showing correlation between the yield of Kharif paddy crop and drought severity based on indices of (a) soil moisture deficit, (b) vegetation health condition, and (c) crop-based water stress at different grid points at 1° resolution across the study area (along with the grid location). (Table form of Fig 5).

| **Grid No.** | **LAT** | **LON** | **SMCI** | **SMDI** | **SWDI-1** | **SWDI-2** | **VCI** | **TCI** | **NDWI** | **SIWSI-1** | **SIWSI-2** |
| --- | --- | --- | --- | --- | --- | --- | --- | --- | --- | --- | --- |
| 1 | 20.5 | 82.5 | 0.70 | 0.27 | 0.06 | - | -0.36 | 0.85 | 0.65 | 0.10 | -0.11 |
| 2 | 19.5 | 82.5 | 0.84 | 0.13 | 0.63 | - | 0.07 | 0.70 | 0.30 | 0.09 | 0.10 |
| 3 | 22.5 | 83.5 | 0.24 | 0.26 | 0.54 | 0.59 | -0.01 | -0.11 | -0.22 | -0.17 | 0.10 |
| 4 | 21.5 | 83.5 | 0.48 | 0.01 | 0.29 | 0.53 | -0.63 | 0.74 | 0.33 | -0.01 | 0.16 |
| 5 | 20.5 | 83.5 | 0.61 | 0.29 | 0.22 | 0.13 | -0.56 | 0.54 | -0.20 | -0.17 | -0.46 |
| 6 | 19.5 | 83.5 | 0.53 | 0.33 | 0.37 | -0.05 | -0.07 | 0.17 | -0.06 | -0.19 | -0.49 |
| 7 | 22.5 | 84.5 | 0.29 | -0.16 | 0.43 | 0.40 | -0.04 | 0.65 | -0.07 | 0.11 | -0.11 |
| 8 | 21.5 | 84.5 | -0.05 | -0.19 | -0.18 | -0.29 | 0.33 | 0.24 | 0.47 | 0.56 | 0.59 |
| 9 | 20.5 | 84.5 | 0.28 | 0.33 | 0.26 | 0.47 | 0.62 | 0.31 | -0.01 | 0.16 | -0.32 |
| 10 | 19.5 | 84.5 | -0.17 | 0.08 | -0.37 | -0.40 | -0.20 | 0.54 | 0.25 | 0.31 | 0.00 |
| 11 | 22.5 | 85.5 | 0.00 | 0.01 | -0.37 | 0.06 | 0.39 | 0.66 | 0.41 | 0.46 | 0.58 |
| 12 | 21.5 | 85.5 | -0.63 | -0.44 | -0.62 | -0.61 | 0.71 | 0.56 | 0.66 | 0.57 | 0.46 |
| 13 | 20.5 | 85.5 | 0.29 | 0.40 | 0.43 | 0.45 | -0.17 | 0.76 | -0.38 | 0.29 | 0.02 |
| 14 | 22.5 | 86.5 | 0.53 | 0.43 | 0.35 | 0.08 | 0.01 | 0.72 | 0.40 | 0.16 | -0.08 |
| 15 | 21.5 | 86.5 | 0.23 | 0.24 | 0.41 | 0.48 | 0.39 | 0.75 | 0.54 | 0.16 | -0.02 |
| 16 | 20.5 | 86.5 | 0.78 | 0.31 | 0.46 | 0.26 | -0.03 | 0.39 | -0.19 | -0.25 | -0.24 |
| 17 | 18.5 | 82.5 | 0.53 | -0.02 | 0.34 | 0.03 | 0.14 | 0.28 | 0.63 | 0.45 | 0.55 |
| 18 | 18.5 | 81.5 | 0.27 | -0.27 | -0.19 | - | -0.50 | 0.02 | 0.03 | 0.04 | -0.42 |

**Table S6.** Table showing correlation between the standardized yield of Kharif paddy crop and drought severity based on indices of (a) soil moisture deficit, (b) vegetation health condition, and (c) crop-based water stress at different grid points at 1° resolution across the study area. (Table form of Figure 6)

| **Grid No.** | **LAT** | **LON** | **SMCI** | **SMDI** | **SWDI-1** | **SWDI-2** | **VCI** | **TCI** | **NDWI** | **SIWSI-1** | **SIWSI-2** |
| --- | --- | --- | --- | --- | --- | --- | --- | --- | --- | --- | --- |
| 1 | 20.5 | 82.5 | 0.69 | 0.22 | 0.05 | - | -0.33 | 0.86 | 0.63 | 0.16 | -0.03 |
| 2 | 19.5 | 82.5 | 0.80 | 0.05 | 0.66 | - | 0.06 | 0.72 | 0.20 | 0.01 | 0.01 |
| 3 | 22.5 | 83.5 | 0.27 | 0.13 | 0.60 | 0.67 | -0.03 | -0.03 | -0.39 | -0.36 | -0.08 |
| 4 | 21.5 | 83.5 | 0.33 | 0.00 | 0.42 | 0.59 | -0.59 | 0.74 | 0.39 | -0.03 | 0.02 |
| 5 | 20.5 | 83.5 | 0.77 | 0.37 | 0.52 | - | -0.57 | 0.57 | -0.30 | -0.24 | -0.44 |
| 6 | 19.5 | 83.5 | 0.48 | 0.26 | 0.38 | -0.06 | -0.03 | 0.35 | 0.06 | -0.02 | -0.34 |
| 7 | 22.5 | 84.5 | 0.28 | -0.10 | 0.44 | 0.35 | 0.07 | 0.77 | -0.12 | -0.06 | -0.16 |
| 8 | 21.5 | 84.5 | 0.05 | -0.18 | -0.26 | -0.36 | 0.26 | 0.33 | 0.41 | 0.52 | 0.58 |
| 9 | 20.5 | 84.5 | 0.25 | 0.32 | 0.22 | 0.49 | 0.51 | 0.44 | -0.17 | 0.02 | -0.40 |
| 10 | 19.5 | 84.5 | -0.16 | 0.01 | -0.45 | -0.48 | -0.13 | 0.67 | 0.13 | 0.18 | -0.12 |
| 11 | 22.5 | 85.5 | -0.11 | -0.08 | -0.52 | -0.11 | 0.52 | 0.80 | 0.54 | 0.54 | 0.66 |
| 12 | 21.5 | 85.5 | -0.59 | -0.44 | -0.69 | -0.65 | 0.69 | 0.51 | 0.66 | 0.56 | 0.44 |
| 13 | 20.5 | 85.5 | 0.29 | 0.40 | 0.43 | 0.45 | -0.17 | 0.76 | -0.38 | 0.29 | 0.02 |
| 14 | 22.5 | 86.5 | 0.51 | 0.40 | 0.35 | 0.07 | 0.01 | 0.70 | 0.37 | 0.12 | -0.11 |
| 15 | 21.5 | 86.5 | 0.23 | 0.24 | 0.41 | 0.48 | 0.39 | 0.75 | 0.54 | 0.16 | -0.02 |
| 16 | 20.5 | 86.5 | 0.42 | 0.14 | 0.66 | 0.24 | 0.15 | 0.55 | 0.38 | 0.39 | 0.37 |
| 17 | 18.5 | 82.5 | 0.60 | -0.02 | 0.36 | 0.06 | 0.13 | 0.30 | 0.66 | 0.40 | 0.46 |
| 18 | 18.5 | 81.5 | 0.27 | -0.27 | -0.19 | - | -0.50 | 0.02 | 0.03 | 0.04 | -0.42 |

**LIST OF SUPPLEMENTARY FIGURES**


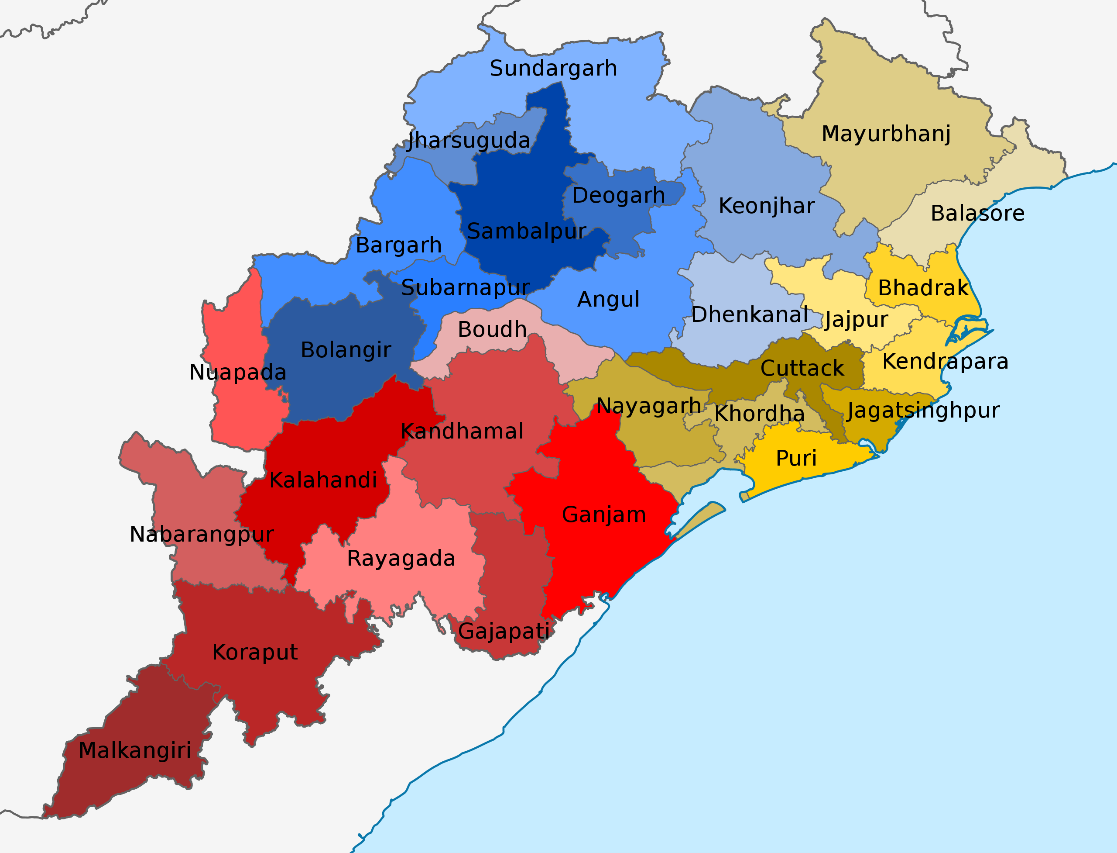


**Figure S1.** Map of Odisha (study area) representing all the States within it.


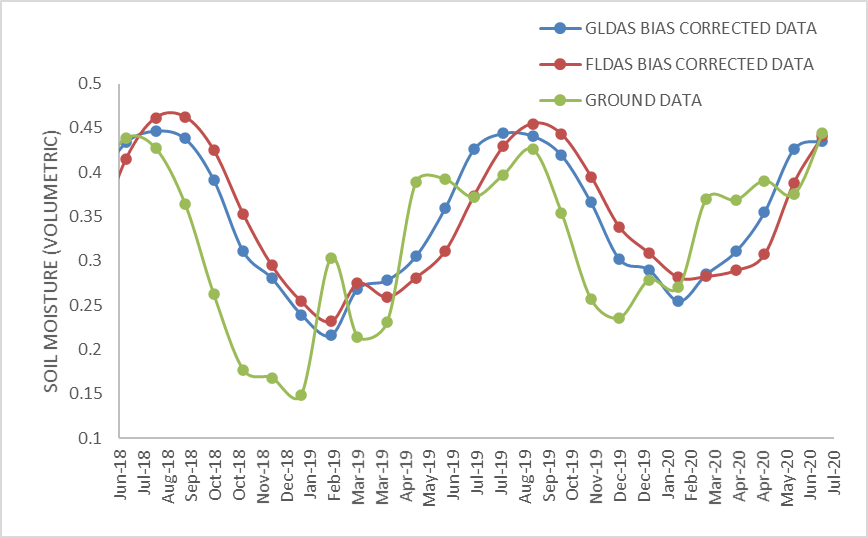


**Figure S2.** Time series of monthly scale soil moisture (m^3^/m^3^) datasets from different sources with bias correction applied at the sample location (21.5° N, 86.5° E).
